# Micro-Arrayed Human Embryonic Stem Cells-Derived Cardiomyocytes for *In Vitro* Functional Assay

**DOI:** 10.1371/journal.pone.0048483

**Published:** 2012-11-12

**Authors:** Elena Serena, Elisa Cimetta, Susi Zatti, Tania Zaglia, Monica Zagallo, Gordon Keller, Nicola Elvassore

**Affiliations:** 1 Industrial Engineering Department, University of Padova, Padova, Italy; 2 Venetian Institute of Molecular Medicine, Padova, Italy; 3 McEwen Centre for Regenerative Medicine, University Health Network, Toronto, Ontario, Canada; Centro Cardiologico Monzino, Italy

## Abstract

**Introduction:**

The heart is one of the least regenerative organs in the body and any major insult can result in a significant loss of heart cells. The development of an *in vitro*-based cardiac tissue could be of paramount importance for many aspects of the cardiology research. In this context, we developed an *in vitro* assay based on human cardiomyocytes (hCMs) and *ad hoc* micro-technologies, suitable for several applications: from pharmacological analysis to physio-phatological studies on transplantable hCMs. We focused on the development of an assay able to analyze not only hCMs viability, but also their functionality.

**Methods:**

hCMs were cultured onto a poly-acrylamide hydrogel with tunable tissue-like mechanical properties and organized through micropatterning in a 20×20 array. Arrayed hCMs were characterized by immunofluorescence, GAP-FRAP analyses and live and dead assay. Their functionality was evaluated monitoring the excitation-contraction coupling.

**Results:**

Micropatterned hCMs maintained the expression of the major cardiac markers (cTnT, cTnI, Cx43, Nkx2.5, α-actinin) and functional properties. The spontaneous contraction frequency was (0.83±0.2) Hz, while exogenous electrical stimulation lead to an increase up to 2 Hz. As proof of concept that our device can be used for screening the effects of pathological conditions, hCMs were exposed to increasing levels of H_2_O_2_. Remarkably, hCMs viability was not compromised with exposure to 0.1 mM H_2_O_2_, but hCMs contractility was dramatically suppressed. As proof of concept, we also developed a microfluidic platform to selectively treat areas of the cell array, in the perspective of performing multi-parametric assay.

**Conclusions:**

Such system could be a useful tool for testing the effects of multiple conditions on an *in vitro* cell model representative of human heart physiology, thus potentially helping the processes of therapy and drug development.

## Introduction

The heart is one of the least regenerative organs in the body [1] and any major insult, due to ischemia, viral infection or other pathologies, can result in a significant loss of heart cells and the progression towards irreversible heart failure. The search for new therapeutic paradigms has become imperative [2] and several lines of research have been investigated [3],[4],[5]. In this context, the development of an *in vitro*-based cardiac tissue could be of paramount importance for many aspects of cardiology research, mainly because of the rapidity of performance, the ease of use and the lower cost of *in vitro* studies compared to *in vivo* ones.

In order to be effective, the new generation *in vitro* assays must overcome some important limitations of actual screening systems, which are mainly based on cytotoxicity measurements of cardiomyocytes randomly plated on a protein coated plastic surface [6]. In particular, new assays should: (*i*) provide information directly related to human cardiac biology and physiology; (*ii*) be highthroughput for fast and low cost screening, (*iii*) integrate a technology able to reproduce defined physio-pathological conditions or precise dosage of drugs; (*iv*) be user friendly.

Several *in vitro* heart models based on artificially engineered cardiac tissue have been proposed, both at the micro- and macro-scale [7],[8],[9],[10],[11],[12],[13],[14]. Despite the originality of these works, they all test animal derived cardiomyocytes and only few *in vitro* cardiac models were developed based on human embryonic stem cell-derived cardiomyocytes (hCMs) [15],[16],[17],[18]. However, all human models were developed at the macro-scale and they all require a high number of cardiomyocytes per construct (4×10^5^ cells minimum). The micro-scale lab on a chip approach would be extremely useful, in addition to the well known advantages of down-scaling [19], reducing the number of hCMs needed, increasing the number of samples per batch and enhancing the high-troughputness of the developed model.

While an animal cell source is very useful, for example during the troubleshooting phase in the development pipeline of a technological device or for basic science research on conserved patho-physiological cardiac mechanisms, the use of hCMs is irreplaceable in sight of a clinical application of the developed device or for specific studies on mechanisms involved in human pathologies. Human and animal cardiology can be quite different, both at the physiological and at the cellular level [20], and such differences can be the cause of withdrawal from the market of several approved drugs. Again, the development of a new therapeutic strategy for cardiac cell therapy or the analysis of a pathological environment (e.g.: inflammation developed by heart failure) on cardiomyocytes should be tested and investigated using hCMs, since these are the type of cells that would be effectively injected in the patient and used in the clinical practice.

In this scenario, the microscaled, highthroughput approach and the use of human samples emerge as the milestones to produce new generation and effective cardiac human *in vitro* models, which would be fairly representative of the human biology and physiology.

In this report, we developed, for the first time to our knowledge, an *in vitro* assay based on hCMs and micro-technologies suitable for several applications: from pharmacological analysis to physio-phatological studies on transplantable hCMs. Our model combines hCMs and micro-scale technologies, adapted to such a sensitive and variable cell source, for multipurpose testing on hCMs under well defined experimental conditions. The hCMs array here developed was designed coupling micro-technology and stem cell engineering to achieve the following features: i) 400 parallel experimental replicates through hCMs micropatterning in array of circular dots (300 µm in diameter) with a consistent and repeatable number of hCMs; ii) elastic substrate with physiological stiffness, able to support hCMs contractions; iii) electrophysiological stimulation assisting the online morphometric analysis of hCMs contractions. We demonstrated that the developed human cardiac assay show functional properties responsive to physio-pathological stimuli. In addition, we gave a proof of concept that it can be used to investigate the effects of a pathological environment on hCMs potentially used in clinics, demonstrating that, besides viability and cytotoxicity, physio-toxicity tests should also be included. By coupling the cell array with a microfluidic platform for selective and compartmentalized delivery of soluble molecules, we highlighted its potential for toxicity and functional study in multi-parametric fashion.

## Materials and Methods

### Human cardiomyocytes derivation and culture

Human cardiomyocytes (hCMs) were derived from HES2 cell line (Wisconsin International Stem Cell Bank) as previously described by Yang et al [21]. Briefly, HES2 colonies were detached from a Matrigel coated dish with collagenase IV (Invitrogen) and trypsin (Invitrogen) and transferred to low adhesive dishes for the EB formation in aggregation medium: basal medium (see Materials and Methods S1) additioned with 10 ng/mL BMP4 (R&D system). From day 1 to day 4 EB were cultured in stage I medium: basal medium (see Materials and Methods S1) with 10 ng/mL BMP4, 5 ng/mL βFGF (R&D system) and 6 ng/mL Activin A (R&D system). EB were then cultured in stage II medium, day 4 to day 8, consisting of basal medium and 10 ng/mL VEGF (R&D system) and 150 ng/mL DKK (R&D system). Finally, from day 8 to day 14, the culture medium consisted of basal medium and 10 ng/mL VEGF and 5 ng/mL βFGF. Cultures were maintained in a 5% CO_2_, 5% O_2_,90% N_2_ environment for the first 14 days and then transferred into a 5% CO_2_ air environment.

### Hydrogel production and protein micropatterning

Poly-acrylamide hydrogel and the micropatterning of adhesion proteins were prepared as previously described [22]–[24]. Briefly, micro-contact printing of adhesion proteins was performed onto the no-fouling surface of poly-acrylamide hydrogel films with an average thickness of 50 µm. Hydrogel films were prepared on chemically modified glass slides 25 mm in diameter polymerizing 20 µL of a 10% acrylamide/bis-acrylamide (Sigma-Aldrich) solution in phosphate-buffered saline (PBS, Gibco-Invitrogen) supplemented with 20 mg/mL photoinitiator (Irgacure 2959; Ciba Specialty Chemicals), by UV light exposure for 3 min (high-pressure mercury vapor lamp (Philips HPR 125 W) emitting at 365 nm). Obtained hydrogel films were purified for 48 hours in ultrapure distilled water to ensure complete removal of the unreacted monomeric units, then allowed to dry and sterilized by exposure to UV light for 20 min.

Adhesion proteins were micropatterned onto the sterilized hydrogel surface in an array of 20×20 circular dots, 300 µm in diameter and 700 µm center-to-center spaced. Protein micropatterning was realized using a PDMS stamp reproducing the desired geometry in relief. The PDMS stamp was inked in the protein solution (mouse laminin 100 µg/mL in PBS, Sigma-Aldrich) for a few seconds and the excess solution was removed. Conformal contact between the dry hydrogel surface and the stamp was then achieved by applying a gentle pressure, thus transferring the protein micropattern onto the substrate. The resulting protein micropattern was homogeneous and no substantial border effect has been observed on protein transfer.

### hCMs microstructured culture

**Figure 1:**
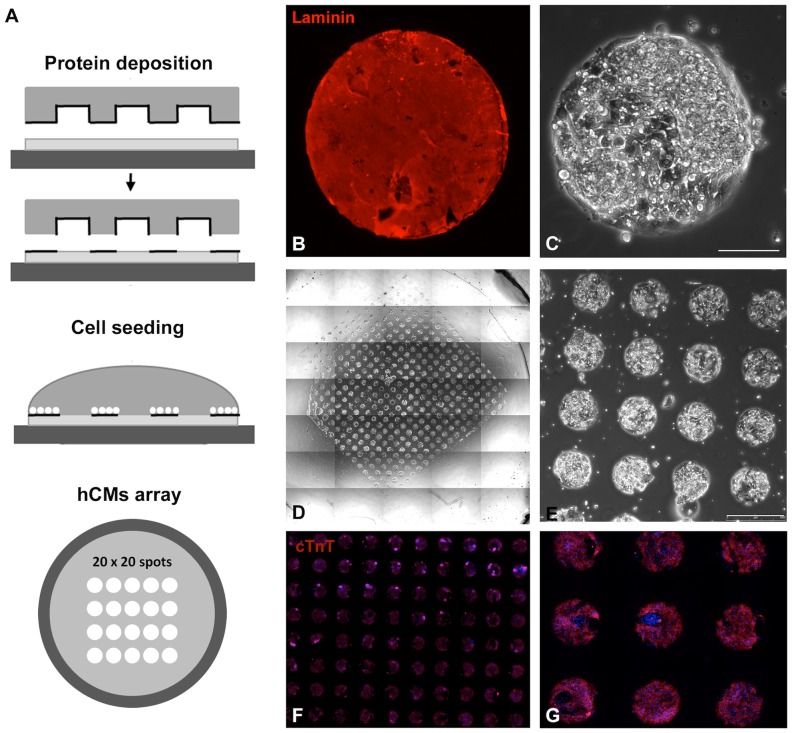
Arrayed hCMs culture. **A** - Schematic representation of hydrogel micropatterning and hCMs seeding. **B** - laminin immunofluorescence of a micropatterned spot. **C–E** - microstructured hCMs culture, **F–G** - cTnT immunofluorescence of the hCMs culture, nuclei were counterstained with hoechst. Scale bars: B–C: 90 µm; E: 500 µm.

Fully differentiated EBs, ranging from 27 to 39 days old, were dissociated to single cells in order to obtain the microstructured hCMs array. EBs were treated with 0.2% Collagenase Type I (Invitrogen) for 45 minutes at 37°C and with trypsin for 5 minutes at 37°C. Trypsin was quenched with Stop solution (50% FCS, 50% IMDM (Invitrogen)). Gentle resuspension of the loosened EBs ensured the obtainment of a single cell suspension. 300 µL of cell suspension (4×10^5^ cells/mL) was dropped over the hydrogel, previously micropatterned (Fig. 1A), in order to obtain an average 300 cells each spot. hCMs were allowed to adhere for 5–8 hours. Cell cultures were kept at 37°C, 5% CO_2_.

### Immunohystochemistry

A standard immunofluorescence protocol was used (see Materials and Methods S1). Primary antibodies were against cardiac troponin T (cTnT, clone 13-11, NeoMarkers), α-actinin (clone 1A4, Sigma Aldrich), connexin 43 (Cx43, clone 4E6.2, Chemicon), Nkx2.5 (clone A-16, Santa Cruz Biotechnology), adult isoforms of cardiac troponin I [25] and adult/fetal isoform of cardiac Troponin T [26] (Ti1 and RVC2 respectively, kindly given by Professor Schiaffino, Padova University). Secondary antibodies used were: goat anti mouse (Invitrogen), goat anti rabbit (Invitrogen), donkey anti goat (Jackson ImmunoLab). Nuclei were counterstained with Hoechst or DAPI (Sigma Aldrich) and samples were mounted with Elvanols, and viewed under a fluorescence microscope.

### Live and Dead assay

Cell viability was evaluated with the LIVE/DEAD assay (Invitrogen). Briefly, hCMs were incubated with 150 µl of 3 µM calcein and 3 µM ethidium bromide in D-PBS (Gibco) for 45 minutes at room temperature. Following incubation, the cells were washed with PBS and labeled cells were observed under a fluorescence microscope.

### Gap-FRAP analysis

In order to evaluate the functional interconnection between hCMs, gap junction functionality was quantitatively determined in living cells by gap-FRAP assay. hCMs were loaded with calcein AM (3 µM for 45 minutes at room temperature, Invitrogen), which permeates gap junction channels. FRAP was performed using a confocal laser scanning microscope (Leica), with an argon laser source at 496 nm. See Materials and Methods S1 for details.

### Morphometric analysis

The morphometric analysis of hCMs contractions were performed coupling the acquisition of contraction displacement by a fast acquisition camera (blue fox, Matrix Vision Gmbh) with a system for electrical stimulation of the culture [27],[28],[29].Despite the possibility to perform morphometric analyses for each single hCMs spot of the array, it is worth to underline that electrophysiological stimulation can not be applied independently for single spots.

We developed a methodology based on the following steps: a) recording of hCMs contractions, b) analysis of the hCMs displacements from the obtained movies, c) representation of the rate of displacements in a 2D graph, d) calculation of the contraction frequency.

The contraction frames were acquired for 5 or 10 seconds every 50 ms. hCMs displacement was analyzed by fixing circular regions of interest (ROI) and analyzing their intensity with the ImageJ Software (version number 1.46 [30]). A minimum of 3 ROI of 2 µm diameter was selected for single cardiac spot. The intensity values were then plotted in a graph showing the intensity, in arbitrary unit, versus time, in seconds. Ten optical fields of 6–8 independent hCMs spots (for a total number of 60–80 independent measurements) were analyzed for each acquired series of frames. The electrical stimulation was applied to the hCMs array using two carbon electrodes (Ladd Research, 3 mm in diameter and 20 mm in length) placed at 10 mm distance and held by a PDMS holder designed to fit a 35 mm Petri dish and to keep electrodes immersed in the culture medium during the analysis.

The electrodes were connected via platinum wires to a function generator (Amel, model 568) programmed to produce a square wave with a 0 V baseline and impulses ranging from 1 V/cm to 6.8 V/cm for 5 ms with frequency ranging from 1 to 4 Hz. A third platinum electrode was inserted to monitor the electrical stimulation by an oscilloscope (LeCroy, LT322). The applied voltage was 6.8 V/cm with a frequency ranging from 1 to 4 Hz and a duration of 5 ms [29].

### Microfluidic platform and array coupling

The multilayered microfluidic platform (overall dimensions: 75×50 mm) was designed for an easy and reversible interface with the cell array. It was fabricated using lithographic techniques and molded in poly-dimethylsiloxane (PDMS) as reported in our recent work [31]. Briefly, the platform is composed of three layers: (i) a medium perfusion layer composed by 8 microfluidic channels (width×height 0.2×0.1 mm) delivering fluids to the culture chamber holding the cell array; (ii) a spacer layer which forms the culture chamber and contains a microfluidic vacuum ring on the bottom; under vacuum it works as a suction pad creating a stable but reversible sealing to the underlying layer; (iii) a support layer for properly housing the cell array: a glass slide. The array integrated in the microfluidic platform is optically accessible. In addition, the reversibly sealing system allows an easy access to the cultured cells. The fluids micro-perfusion inside the platform is allowed connecting channels to two syringe pumps (PHD, Harvard Apparatus, Holliston, MA) through tygon tubings (Cole Palmer, USA). Flow rate used in the reported experiments was 1 µl/min. At this hydrodynamic condition, the compartimentalization of soluble environment can be achieved [31]. As proof of concept, we used murine myoblast cell line (C2C12, ATCC), cultured as previously reported [24] for short and long term compartmentalization of biological responses.

## Results

### Micropatterned hCMs array characterization

**Figure 2:**
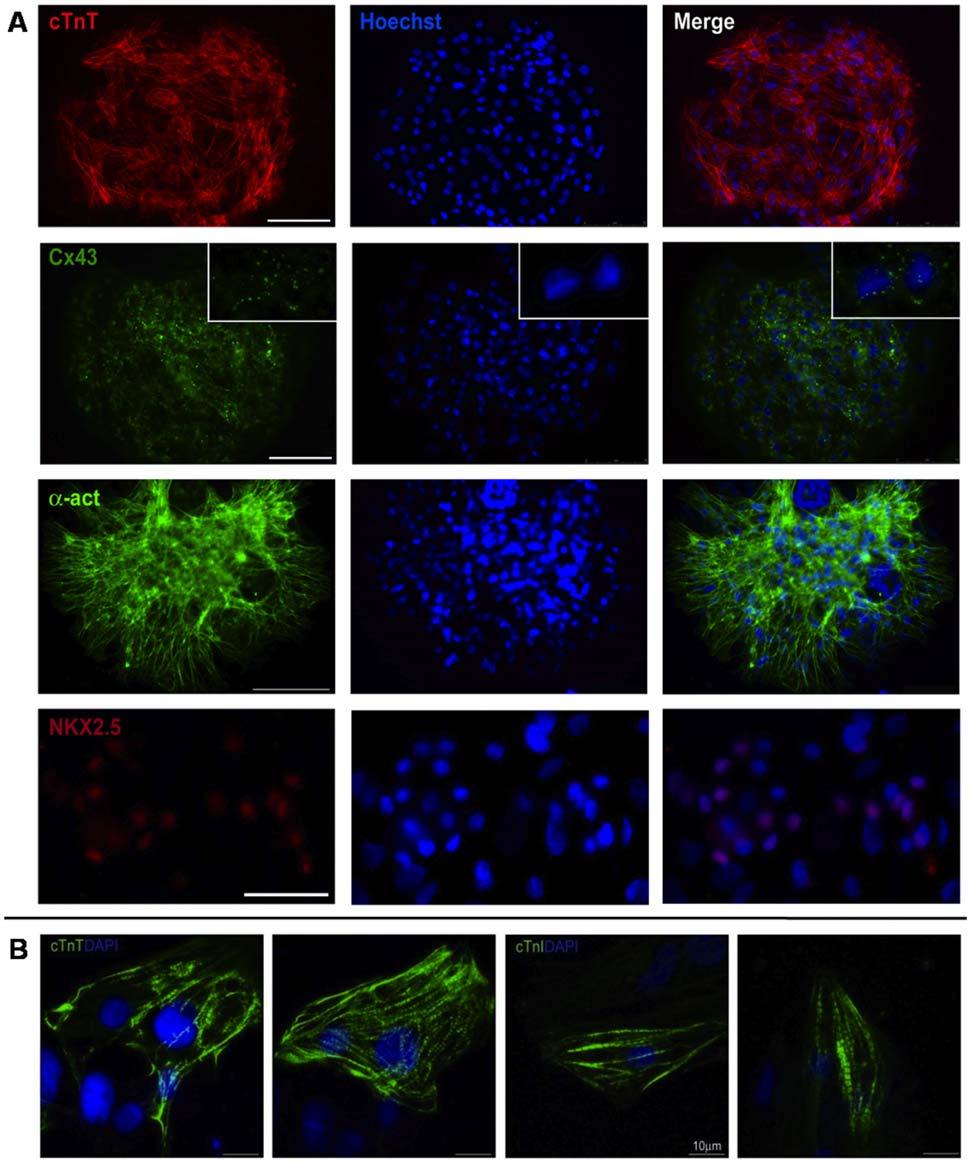
Characterization of microstructured hCMs culture. **A** - Cardiac markers immunofluorescence; cTnT, connexin 43, α-actinin, NKX2.5. Nuclei were counterstained with hoechst. **B** - Immunofluorescence against and adult isoform of cardiac Troponin I of T39 hCMs and adult/fetal isoform of cardiac Troponin T of T39 hCMs, nuclei were counterstained with hoechst. Scale bars: A: 75 µm and 25 µm for NKX2.5; B: 10 µm.

hCMs were obtained from the human embryonic stem cell line HES2, following the protocol described by Yang and colleagues [21]. The hCMs characterization, at the end of the differentiation protocol, has been previously reported by the same group. Recently, the mechanical environment of cell culture has captured increasing interest among researchers, especially in the field of stem cell expansion and differentiation [32]. It has been demonstrated that the development of sarcomeric structures of human striated muscles [22] and the contractile functionality of chicken cardiomyocytes [33] are influenced by the substrate stiffness. For these reasons, we did not culture hCMs onto standard rigid Petri dishes or glass coverslips, but on a soft poly-acrylamide (PA) hydrogel (elastic modulus: 15 kPa). The micropatterned hydrogel substrates, specifically tailored for contracting muscle cells, were designed and developed in accordance with our previous works [22],[23] (Fig. 1, A). After EB disgregation and cell seeding onto the microstructured hydrogel, the culture resulted in a 20×20 array of cellularized circular spots defined with micrometric precision (Fig. 1, B–E). After adhesion, we verified a fair distribution of the cell number of in each spot by counting DAPI stained nuclei: 260 cells/spot with an average variation of 15% was obtained. Moreover, the percentage of cardiomyocytes per spot was around 90% (based on the percentage of cardiac Troponin T positive cells), with a fair distribution on the entire array (Fig. 1, F–G). The expression of cardiac Troponin T (cTnT) was maintained for several days (up to 7 days). We will thus refer to this culture as hCMs. The maintenance of cardiac markers on micropatterned cells was verified after 5–7 days of culture onto the hydrogel (Fig. 2A). cTnT, Cx43, α-actinin and Nkx2.5 were analyzed. At this time point, spontaneous and electrically induced contractions were observed (Movie S1). In addition, the expression of adult isoforms of cardiac Troponin I and T (Fig. 2B) was observed in our hCMs derived from EBs older than 25 days and cultured onto micropatterned hydrogel for at least 6 days.

Gap-FRAP experiments have been performed to test the functionality of hCMs gap junctions [34] (Figure S1). Confocal microscopy allowed the recording of the target cell's fluorescence recovery on a single plan before and after the photobleaching by scanning argon laser beam (Figure S1 A–B). Fluorescence recovery (Figure S1 C) is related to dye transport across gap junction. Table S1 reports the fitting parameters, k and A, of the fitting recovery curves (Figure S1 D). Results of the fitting showed the presence of functional gap junctions in hCMs. The percentage of recovery for control cells was about 5 times smaller than the percentage calculated for hCMs.

In order to further optimize the developed hCMs array, we investigated the optimal substrate stiffness for hCMs culture. In particular, it has been reported that chicken cardiomyocytes contraction is inhibited when cultured onto a substrate whose stiffness ranges from 35 to70 kPa. Such a range of substrate elasticity is representative of the non-contractile fibrotic tissue formed after a myocardial infarction, while a normal myocardium has an elastic modulus of E ≈ 10 kPa [33]. The elastic modulus of the PA hydrogel was easily tuned by varying the composition of the pre-polymer solution, as previously reported [22]. hCMs microstructured cultures were performed on a 20% PA hydrogel with E ≈ 35 kPa, in order to mimic the fibrotic tissue. After 7 days of culture we evaluated hCMs viability (live and dead assay) and contractility (Figure S2). We didn't observe any significant difference between hCMs cultured on 15 kPa and 35 kPa hydrogels, in terms of viability (data not shown), functional properties (Figure S2, A and C) and sarcomeric organization of cTnT (Figure S2, B and D). This is likely due to the multilayer cellular growth of hCMs over the hydrogel, which hampered the substrate stiffness effects. Therefore, we proceeded by using 15 kPa hydrogels.

### Morphometric analysis of hCMs array

Once the cardiac phenotype of the hCMs micropatterned onto the soft substrate had been verified, we moved our investigations to the biophysical properties of these cells. We observed that the spontaneous contractile activity of arrayed hCMs was synchronous intra each spot, additional proof of functional cell-cell interactions, while the contractions become non-synchronous inter-spots. We therefore decided to couple exogenous electrical stimulation in order to verify hCMs capability to respond to external stimuli (Excitation-Contraction coupling) and for synchronizing the contractions of the spots (evaluating the maximum contraction frequency).

**Figure 3:**
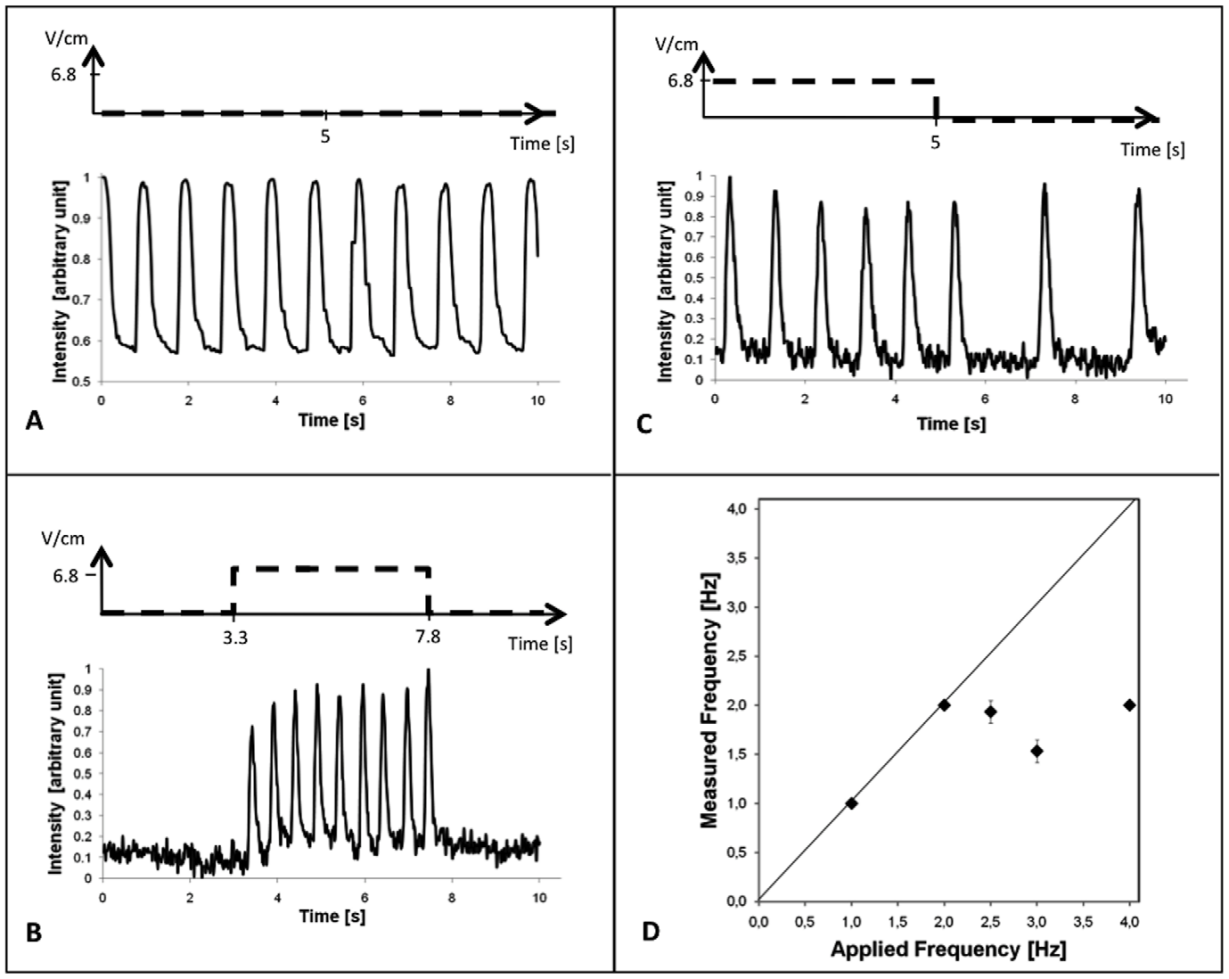
Experimental validation of morphometric analysis. **A** - Morphometric analysis of spontaneous contractions. **B** - Morphometric analysis of hCMs induced to contract with electrical stimulations. **C** - Morphometric analysis of spontaneously contracting hCMs captured by exogenous electrical stimulations. In graphs A–C, the dashed line represents the applied exogenous electrical stimulation, the black line shows the contractions/displacement of hCMs. **D** - Graph reporting the measured frequency as a function of the applied frequency.

Micropatterned hCMs showed some spontaneous beating (Fig. 3A), and they were induced to contract with exogenous electrical stimulation (Fig. 3B). We were able to monitor hCMs contractions and calculate their frequency (Fig. 3).The robustness of the developed methodology is shown in figures 3B and C. Figure 3B shows the contraction traces obtained with an off-on-off sequence of electrical stimulation: 0 V/cm; 6.8 V/cm at 2 Hz for 4.5 s; 0 V/cm (dashed line). Spontaneous contractions were not present in the absence of the electrical field, while during electrical stimulation hCMs are induced to contract and paced to a frequency of 2 Hz. Similarly, figure 3C shows a contraction-graph of spontaneously contracting hCMs. The applied stimulation, 6.8 V/cm and 1 Hz frequency, stops after 5 s. The obtained graph (Fig. 3C) clearly shows that hCMs are paced during the application of an exogenous electrical stimulation (from 1 to 5 s their contraction frequency is 1 Hz), while after the stimulation they return to a spontaneous contraction frequency of 0.6 Hz.

### Application of the *in vitro* model: pathological conditions assay

**Figure 4:**
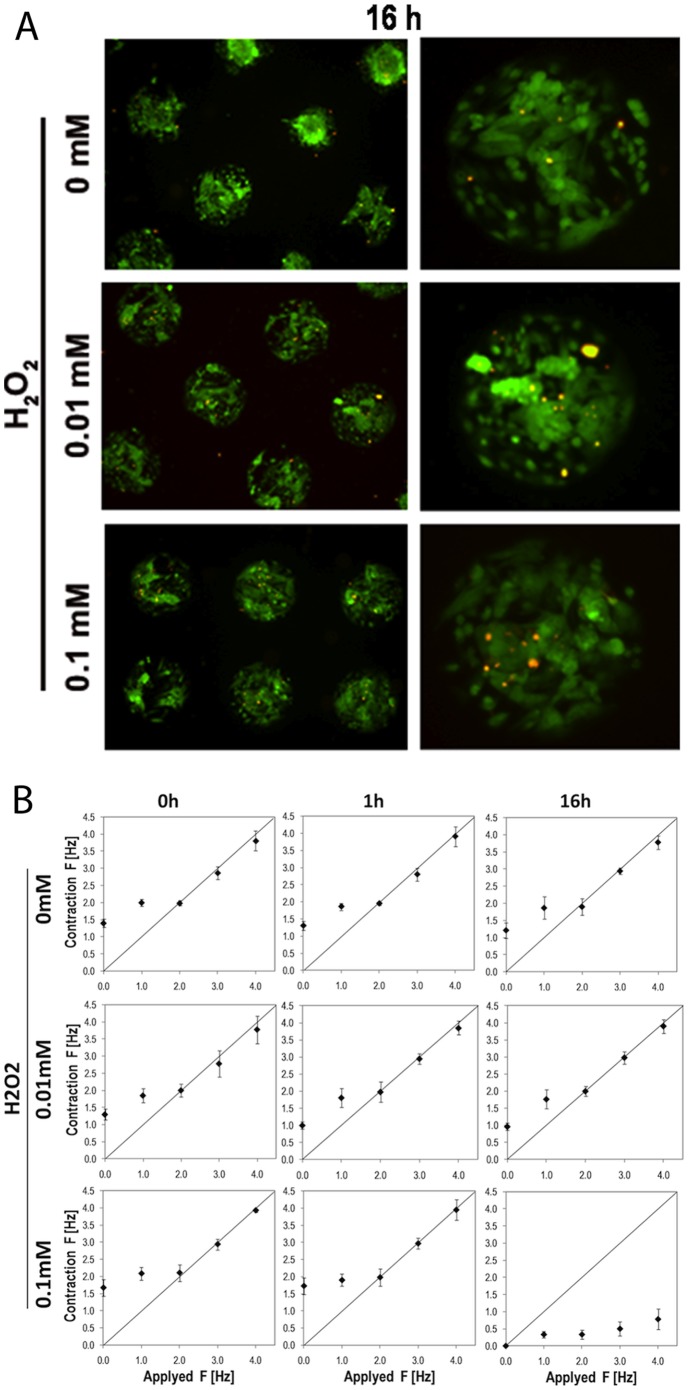
Effects of oxidative stress on hCMs. **A** - Live and dead analysis after 16 hours of exposure to 0 mM, 0.01 mM and 0.1 mM H_2_O_2_. **B** - Mophometric analisis of hCMs exposed to 0 mM, 0.01 mM and 0.1 mM H_2_O_2_ for 0, 1 and 16 hours.

In view of giving a proof of concept that our system could be used as an *in vitro* model for testing the effects of a pathological environment on the functional properties of transplantable human cardiomyocytes, we exposed hCMs to hydrogen peroxide (H_2_O_2_) to mimic the oxidative stress implicated in various disease states. In fact, reactive oxygen species are generated during both ischemia and reperfusion phases [35] and the inflammatory environment of a healing infarct could present high levels of oxygen-free radicals [36]. We thus exposed micropatterned hCMs to increasing levels of H_2_O_2_ (0; 0.01 and 0.1 mM) for 1 and 16 hours, in accordance with other studies [37]. hCMs viability and functional properties were analyzed. We observed that hCMs viability after 1 and 16 hours was not affected by any of the H_2_O_2_ concentrations tested (Fig. 4A). The percentage of dead cells (Live and Dead test) were (4.5±1)%in control, (3±1)% in samples treated with 0.01 mM H_2_O_2_ and (3.5±1)% in hCMs treated with 0.1 mM H_2_O_2_. Positive control of hydrogen peroxide citotoxicity was performed using 0.5 mM H_2_O_2_, showing 100% of dead hCMs (data not shown). Interestingly the contractility and E-C coupling capability were maintained for all conditions, after short time exposition (1 hour). On the other hand, the highest concentration of H_2_O_2_ (0.1 mM) applied for 16 hours (Fig. 4B) suppressed the functional properties of hCMs (contractility) while maintaining their viability. This experimental observation was confirmed with an average of 50 individual spots and 3 independent analyses.

### Microfluidic platform for multi-parametric assay

**Figure 5:**
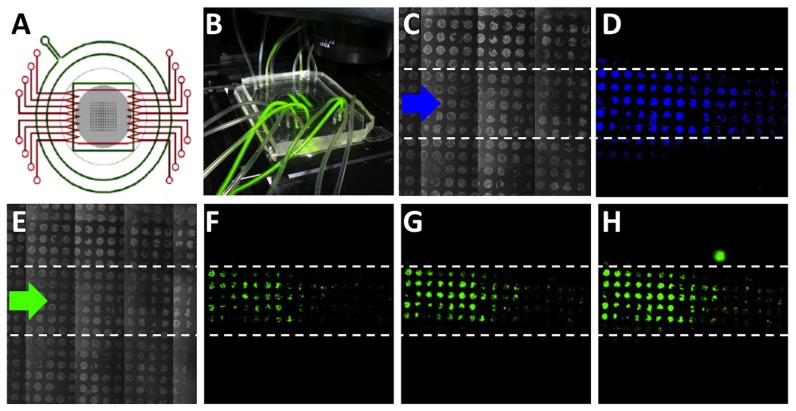
Coupling of the cell array with a microfluidic platform. **A** - Schematic representation of the microfluidic platform containing 8 microfluidic channels for media perfusion (in red) and containing a membrane-vacuum system (in green), acting as a suction pad for reversible sealing and which delimits the culture chamber. Cell array is represented in grey. **B** - Images of the assembled microfluidic platform under fluorescent light on the microscope stage. Two channels deliver a fluorescein solution. **C–D** - Validation of the coupled system using a nuclear dye (HOECHST). Phase contrast (C) and fluorescence image (D) of the entire cell array show how the HOECHST signal could be detected only on the area selectively exposed to the fluid stream containing the nuclear dye (blue arrows). **E–H** - Validation of the coupled system using adenoviral vectors for EGFP delivery. (E) Phase contrast of the entire cell array and (F–H) fluorescence images of the temporal sequence showing an increased EFGP expression at 16 h (F), 22 h (G), 26 h (H) post-infection. The viral transduction is clearly compartmentalized on the area selectively exposed to the fluid stream containing the viral particles.

In the perspective of using the developed *in vitro* model for highthroughput screening of multiple stimuli or drugs, we developed, as proof of concept, an *ad-hoc* system for a selective and compartmentalized perfusion of a myoblast cell array. Perfusion was established using a microfluidic platform (Figure 5A, B), as reported in our recent work [31]. The microfluidic experimental setup and perfusion flow rate allow a strict control over the transport regimes and the establishment of spatial-temporal defined compartment of soluble environment over the cell array.

As proof of concept, we supply experimental evidence that short and long term compartmentalized biological stimulations can be successfully provided. First of all, the compartmentalization of biochemical stimulation was verified by perfusing (1 µl/min flow rate) the cell array with two fluid streams containing HOECHST nuclear dye at 10 µg/ml for 10 minutes. The nuclear fluorescent signal was detected only on the spots selectively exposed to the corresponding fluid stream (Fig. 5C, D). Furthermore, in order to validate the possibility to obtain long-term compartmentalized biological process, the different areas of the cell array were perfused with medium additioned with adenoviral vectors for EGFP delivery at MOI 50 for 26 hours. As result, EGFP was selectively expressed only on the dots exposed to the fluid stream containing the viral particles (Fig. 5E–H). An increasing compartmentalized EGFP expression, consistent with the areas exposed to viral particles, was observed at long-term time points (16, 22 and 26 hours).

## Discussion

In this work we developed, for the first time to our knowledge, an *in vitro* model based on human cardiomyocytes and *ad hoc* micro-technologies for physio-pathological studies.

Usually, *in vitro* studies involving hCMs use clusters of cardiomyocytes derived by the dissection of the EB's contracting area or by co-culture with other cell types [38],[39]. Here, we report the development of a microstructured array of hCMs (20×20 spots, with a high number of hCMs per spot) where each microstructured hCMs spot could represent an independent sample deriving from the same batch of cells. Consequently, the array offers the possibility of analyzing different spots at the same time, exposed to identical conditions, with a consistent/repeatable number of hCMs per spot. The developed *in vitro* model could thus give a high number of output information per experiment and reduce the high variability usually observed when working with a primary human cell source [40].

We did not underestimate the fact that hESC-derived cardiomyocytes are not fully representative of adult cardiomyocytes: the differentiation stage of hCMs is a crucial point, within the aim of developing an *in vitro* model of human cardiac tissue.

Despite further analyses to asses hESC-derived cardiomyocytes functionality should be performed in the perspective of using the array in a pre-clinical trial, we evaluate (i) the expression of adult isoforms of cardiac Troponin T and I and (ii) the gap-junction interconnectivity of the obtained hCMs sposts. It is worth underlining that, further to the maintenance of major cardiac markers (cTnT, Cx43, α-actinin and Nkx2.5), the expression of adult isoforms of cardiac Troponin I and T (Fig. 2B) were observed in our hCMs. In addition, with the goal of obtaining a higher-hierarchy functional tissue, rather than clusters of single scarcely communicating cardiac cells, gap-FRAP analyses have been performed. Such experiments showed that cultured hCMs have gap functionality and therefore proves their functional interconnection within the patterned spot.

The development of a morphometric assay to detect hCMs spontaneous and induced contractions, allows a functional characterization of the array. The morphometric analysis has been developed based on a methodology widely accepted by researchers in this field: imaging analyses of contracting areas. For instance, studying contracting cells or tissues by video recording and digitalizing of the captured images has been applied for contractions of both three-dimensional tissue constructs [11],[27],[41],[29],[42]and two-dimensional cell cultures [43],[44],[45]. We improved the analysis through the use of micropatterning: each hCMs spot has clear and defined edges, and so contracting cardiomyocytes are detected and analyzed with higher accuracy. Application of an exogenous electrical stimulation results in inter-spot pacing to a frequency of 2 Hz, demonstrating hCMs capability to respond to external stimuli.

As proof of concept that the developed *in vitro* model can be used for screening the effects of pathological conditions, the hCMs array was exposed to increasing levels of H_2_O_2_. Remarkably, low level of oxidative stress is ineffective for hCMs viability, while it could indeed dramatically compromise their functionality, in terms of contractile ability. The results obtained with hCMs are in accordance with recent data reported for murine skeletal muscles and rat cardiomyocytes. In particular, rat cardiomyocytes exposed to 0.2 mM H_2_O_2_ start showing modifications in the calcium handling as soon as 90 s after treatment. As recently suggested [46],[47], the suppression of hCMs contractility after H_2_O_2_ treatment, observed in this work, could derive from the interaction between reactive oxygen species (ROS) and Ca^2+^ handling machinery and myofibrils. This observation could be relevant for rational understating why, after *in vivo* injection into compromised or damaged heart, the surviving cells do not integrate or have a poor functional integration with the host tissue. In this scenario, our assay could potentially be used to investigate *in vitro* the best preconditioning strategy or pro-survival factors, which can increase the number of surviving and integrating hCMs after transplantation [1] into a diseased heart. Taken together, the results clearly indicate that an *in vitro* test merely based on cell toxicity could give misleading outcomes, especially in the case of cardiac drug or therapy development. Such a physiotoxicity test, analyzing physiology in parallel to cytotoxicity, is thus required in order to have a complete and reliable set of data.

A microfluidic platform was also coupled with the cell array. This platform was validated for the selective and compartmentalized delivery of soluble stimuli on different spots of the array. Those preliminary results are extremely promising in sight of the following applications of the system. For example, it would be possible to selectively treat neighboring areas of the hCMs array with different drugs or to simultaneously evaluate the effects of a range of concentrations of a defined substance. This would prove extremely useful for toxicological studies.

The developed array of micropatterned hCMs lead us to gain repeatability and robustness, and allowed multiple analyses per batch of cells. This study demonstrates the feasibility of developing an *in vitro* based test for hCMs, able to give insight on both viability and functionality of hCMs. Secondly, we demonstrated that collecting information about the functional properties of hCMs could give a clearer scenario about possible secondary physiological effects of a pathological environment (e.g.: oxidative stress). Importantly, this study emphasizes the importance of multiple readouts from an *in vitro* model, both in terms of the number of data acquired in parallel (array of 20×20 spots) and in terms of the type of analysis (citotoxicity and functional contractions).

## Supporting Information

Materials and Methods S1 Supplementary information about hCMs culture medium, immunohystochemistry and Gap-FRAP analysis.
(PDF)

Figure S1 **Gap-FRAP analysis.** **A–C** - Representative images of fluorescence restoration in hCMs, target cell is indicated by an arrow, scale bar: 50 µm. **A** - Intensity of calceine AM fluorescence before photobleaching. **B** - Fluorescence right after photobleaching. **C** -Fluorescence recovery after 7.5 minutes. **D** - Graph representing the kinetic profiles of raw and fitted recovery data.
(TIF)

Figure S2 **Effects of susbtrate stiffness on hCMs.A, B** - Mophometric analysis and cTnT immunofluorescence of hCMs cultured onto 15 kPa. C, **D** - Mophometric analysis and cTnT immunofluorescence of hCMs cultured onto a 35 kPa hydrogel. Nuclei were counterstained with hoechst.
(TIF)

Movie S1 **Micropattered contracting hCMs.**
(MP4)

Table S1 **Gap-FRAP analysis.** The table reports the value of A and k.
(DOCX)

## References

[1] Laflamme MA, Chen KY, Naumova AV, Muskheli V, Fugate JA, et al (2007) Cardiomyocytes derived from human embryonic stem cells in pro-survival factors enhance function of infarcted rat hearts. Nat Biotech 25: 1015–1024 doi:10.1038/nbt1327 10.1038/nbt1327 17721512

[2] Habib M, Caspi O, Gepstein L (2008) Human embryonic stem cells for cardiomyogenesis. J Mol Cell Cardiol 45: 462–474 doi:10.1016/j.yjmcc.2008.08.008 18775434 10.1016/j.yjmcc.2008.08.008

[3] Freund C, Mummery CL (2009) Prospects for pluripotent stem cell-derived cardiomyocytes in cardiac cell therapy and as disease models. J Cell Biochem 107: 592–599 doi:10.1002/jcb.22164 19449339 10.1002/jcb.22164

[4] Shin DD, Brandimarte F, De Luca L, Sabbah HN, Fonarow GC, et al (2007) Review of current and investigational pharmacologic agents for acute heart failure syndromes. Am J Cardiol 99: 4A–23A doi:10.1016/j.amjcard.2006.11.025 10.1016/j.amjcard.2006.11.025 17239703

[5] Wang F, Guan J (2010) Cellular cardiomyoplasty and cardiac tissue engineering for myocardial therapy. Adv Drug Deliv Rev 62: 784–797 doi:10.1016/j.addr.2010.03.001 20214939 10.1016/j.addr.2010.03.001

[6] Möller C, Slack M (2010) Impact of new technologies for cellular screening along the drug value chain. Drug Discov Today 15: 384–390 doi:10.1016/j.drudis.2010.02.010 20206290 10.1016/j.drudis.2010.02.010

[7] Katare RG, Ando M, Kakinuma Y, Sato T (2010) Engineered heart tissue: a novel tool to study the ischemic changes of the heart in vitro. PLoS ONE 5: e9275 doi:10.1371/journal.pone.0009275 20174664 10.1371/journal.pone.0009275 PMC2822866

[8] Hansen A, Eder A, Bönstrup M, Flato M, Mewe M, et al (2010) Development of a drug screening platform based on engineered heart tissue. Circ Res 107: 35–44 doi:10.1161/CIRCRESAHA.109.211458 20448218 10.1161/CIRCRESAHA.109.211458

[9] Engelmayr GC Jr, Cheng M, Bettinger CJ, Borenstein JT, Langer R, et al (2008) Accordion-like honeycombs for tissue engineering of cardiac anisotropy. Nat Mater 7: 1003–1010 doi:10.1038/nmat2316 18978786 10.1038/nmat2316 PMC2613200

[10] Masuda S, Shimizu T, Yamato M, Okano T (2008) Cell sheet engineering for heart tissue repair. Adv Drug Deliv Rev 60: 277–285 doi:10.1016/j.addr.2007.08.031 18006178 10.1016/j.addr.2007.08.031

[11] Radisic M, Park H, Shing H, Consi T, Schoen FJ, et al (2004) Functional assembly of engineered myocardium by electrical stimulation of cardiac myocytes cultured on scaffolds. Proc Natl Acad Sci USA 101: 18129–18134 doi:10.1073/pnas.0407817101 15604141 10.1073/pnas.0407817101 PMC539727

[12] Song H, Yoon C, Kattman SJ, Dengler J, Massé S, et al (2010) Interrogating functional integration between injected pluripotent stem cell-derived cells and surrogate cardiac tissue. Proc Natl Acad Sci USA 107: 3329–3334 doi:10.1073/pnas.0905729106 19846783 10.1073/pnas.0905729106 PMC2840418

[13] Grosberg A, Alford PW, McCain ML, Parker KK (2011) Ensembles of engineered cardiac tissues for physiological and pharmacological study: Heart on a chip. Lab on a Chip. Available:http://www.ncbi.nlm.nih.gov/pubmed/22072288. Accessed 23 November 2011. 10.1039/c1lc20557a PMC4038963 22072288

[14] Kim SB, Bae H, Cha JM, Moon SJ, Dokmeci MR, et al (2011) A cell-based biosensor for real-time detection of cardiotoxicity using lensfree imaging. Lab Chip 11: 1801–1807 doi:10.1039/c1lc20098d 21483937 10.1039/c1lc20098d PMC3611966

[15] Shapira-Schweitzer K, Habib M, Gepstein L, Seliktar D (2009) A photopolymerizable hydrogel for 3-D culture of human embryonic stem cell-derived cardiomyocytes and rat neonatal cardiac cells. J Mol Cell Cardiol 46: 213–224 doi:10.1016/j.yjmcc.2008.10.018 19027751 10.1016/j.yjmcc.2008.10.018

[16] Caspi O, Lesman A, Basevitch Y, Gepstein A, Arbel G, et al (2007) Tissue engineering of vascularized cardiac muscle from human embryonic stem cells. Circ Res 100: 263–272 doi:10.1161/01.RES.0000257776.05673.ff 17218605 10.1161/01.RES.0000257776.05673.ff

[17] Tulloch NL, Muskheli V, Razumova MV, Korte FS, Regnier M, et al (2011) Growth of engineered human myocardium with mechanical loading and vascular coculture. Circ Res 109: 47–59 doi:10.1161/CIRCRESAHA.110.237206 21597009 10.1161/CIRCRESAHA.110.237206 PMC3140796

[18] Schaaf S, Shibamiya A, Mewe M, Eder A, Stöhr A, et al (2011) Human engineered heart tissue as a versatile tool in basic research and preclinical toxicology. PLoS ONE 6: e26397 doi:10.1371/journal.pone.0026397 22028871 10.1371/journal.pone.0026397 PMC3197640

[19] Hong J, Edel JB, deMello AJ (2009) Micro- and nanofluidic systems for high-throughput biological screening. Drug Discovery Today 14: 134–146 doi:10.1016/j.drudis.2008.10.001 18983933 10.1016/j.drudis.2008.10.001

[20] Bers DM (2002) Cardiac excitation-contraction coupling. Nature 415: 198–205 doi:10.1038/415198a 11805843 10.1038/415198a

[21] Yang L, Soonpaa MH, Adler ED, Roepke TK, Kattman SJ, et al (2008) Human cardiovascular progenitor cells develop from a KDR+ embryonic-stem-cell-derived population. Nature 453: 524–528 doi:10.1038/nature06894 18432194 10.1038/nature06894

[22] Serena E, Zatti S, Reghelin E, Pasut A, Cimetta E, et al (2010) Soft substrates drive optimal differentiation of human healthy and dystrophic myotubes. Integr Biol (Camb) 2: 193–201 doi:10.1039/b921401a 20473399 10.1039/b921401a

[23] Cimetta E, Pizzato S, Bollini S, Serena E, De Coppi P, et al (2009) Production of arrays of cardiac and skeletal muscle myofibers by micropatterning techniques on a soft substrate. Biomed Microdevices 11: 389–400 doi:10.1007/s10544-008-9245-9 18987976 10.1007/s10544-008-9245-9

[24] Zatti S, Zoso A, Serena E, Luni C, Cimetta E, et al (2012) Micropatterning Topology on Soft Substrates Affects Myoblast Proliferation and Differentiation. Langmuir 28: 2718–2726 doi:10.1021/la204776e 22217143 10.1021/la204776e

[25] Saggin L, Gorza L, Ausoni S, Schiaffino S (1989) Troponin I switching in the developing heart. J Biol Chem 264: 16299–16302. 2777792

[26] Saggin L, Ausoni S, Gorza L, Sartore S, Schiaffino S (1988) Troponin T switching in the developing rat heart. J Biol Chem 263: 18488–18492. 2973462

[27] Tandon N, Cannizzaro C, Chao P-HG, Maidhof R, Marsano A, et al (2009) Electrical stimulation systems for cardiac tissue engineering. Nat Protoc 4: 155–173 doi:10.1038/nprot.2008.183 19180087 10.1038/nprot.2008.183 PMC2775058

[28] Serena E, Flaibani M, Carnio S, Boldrin L, Vitiello L, et al (2008) Electrophysiologic stimulation improves myogenic potential of muscle precursor cells grown in a 3D collagen scaffold. Neurol Res 30: 207–214 doi:10.1179/174313208X281109 18397614 10.1179/174313208X281109

[29] Cannizzaro C, Tandon N, Figallo E, Park H, Gerecht S, et al (2007) Practical aspects of cardiac tissue engineering with electrical stimulation. Methods Mol Med 140: 291–307. 18085215 10.1007/978-1-59745-443-8_16

[30] Available: http://rsbweb.nih.gov/.

[31] Cimetta E, Franzoso M, Trevisan M, Serena E, Zambon A, et al (2012) Microfluidic-driven viral infection on cell cultures: Theoretical and experimental study. Biomicrofluidics 6 doi:10.1063/1.4723853 10.1063/1.4723853 PMC3382339 23734169

[32] Engler AJ, Sen S, Sweeney HL, Discher DE (2006) Matrix elasticity directs stem cell lineage specification. Cell 126: 677–689 doi:10.1016/j.cell.2006.06.044 16923388 10.1016/j.cell.2006.06.044

[33] Engler AJ, Carag-Krieger C, Johnson CP, Raab M, Tang H-Y, et al (2008) Embryonic cardiomyocytes beat best on a matrix with heart-like elasticity: scar-like rigidity inhibits beating. J Cell Sci 121: 3794–3802 doi:10.1242/jcs.029678 18957515 10.1242/jcs.029678 PMC2740334

[34] Giaume C (2001) Connexin methods and protocols. Humana Press. 504 p.

[35] Vanden Hoek TL, Li C, Shao Z, Schumacker PT, Becker LB (1997) Significant levels of oxidants are generated by isolated cardiomyocytes during ischemia prior to reperfusion. J Mol Cell Cardiol 29: 2571–2583 doi:10.1006/jmcc.1997.0497 9299379 10.1006/jmcc.1997.0497

[36] Robey TE, Saiget MK, Reinecke H, Murry CE (2008) Systems approaches to preventing transplanted cell death in cardiac repair. J Mol Cell Cardiol 45: 567–581 doi:10.1016/j.yjmcc.2008.03.009 18466917 10.1016/j.yjmcc.2008.03.009 PMC2587485

[37] Cook SA, Sugden PH, Clerk A (1999) Regulation of bcl-2 family proteins during development and in response to oxidative stress in cardiac myocytes: association with changes in mitochondrial membrane potential. Circ Res 85: 940–949. 10559141 10.1161/01.res.85.10.940

[38] Braam SR, Tertoolen L, van de Stolpe A, Meyer T, Passier R, et al (2010) Prediction of drug-induced cardiotoxicity using human embryonic stem cell-derived cardiomyocytes. Stem Cell Res 4: 107–116 doi:10.1016/j.scr.2009.11.004 20034863 10.1016/j.scr.2009.11.004

[39] Jonsson MKB, Duker G, Tropp C, Andersson B, Sartipy P, et al (2010) Quantified proarrhythmic potential of selected human embryonic stem cell-derived cardiomyocytes. Stem Cell Res 4: 189–200 doi:10.1016/j.scr.2010.02.001 20303332 10.1016/j.scr.2010.02.001

[40] Pera MF, Tam PPL (2010) Extrinsic regulation of pluripotent stem cells. Nature 465: 713–720 doi:10.1038/nature09228 20535200 10.1038/nature09228

[41] Radisic M, Fast VG, Sharifov OF, Iyer RK, Park H, et al (2009) Optical mapping of impulse propagation in engineered cardiac tissue. Tissue Eng Part A 15: 851–860 doi:10.1089/ten.tea.2008.0223 18847360 10.1089/ten.tea.2008.0223 PMC2759871

[42] Radisic M, Yang L, Boublik J, Cohen RJ, Langer R, et al (2004) Medium perfusion enables engineering of compact and contractile cardiac tissue. Am J Physiol Heart Circ Physiol 286: H507–516 doi:10.1152/ajpheart.00171.2003 14551059 10.1152/ajpheart.00171.2003

[43] Dolnikov K, Shilkrut M, Zeevi-Levin N, Gerecht-Nir S, Amit M, et al (2006) Functional properties of human embryonic stem cell-derived cardiomyocytes: intracellular Ca2+ handling and the role of sarcoplasmic reticulum in the contraction. Stem Cells 24: 236–245 doi:10.1634/stemcells.2005–0036 16322641 10.1634/stemcells.2005-0036

[44] Au HTH, Cheng I, Chowdhury MF, Radisic M (2007) Interactive effects of surface topography and pulsatile electrical field stimulation on orientation and elongation of fibroblasts and cardiomyocytes. Biomaterials 28: 4277–4293 doi:10.1016/j.biomaterials.2007.06.001 17604100 10.1016/j.biomaterials.2007.06.001 PMC2039774

[45] Heidi Au HT, Cui B, Chu ZE, Veres T, Radisic M (2009) Cell culture chips for simultaneous application of topographical and electrical cues enhance phenotype of cardiomyocytes. Lab Chip 9: 564–575 doi:10.1039/b810034a 19190792 10.1039/b810034a

[46] Lamb GD, Westerblad H (2011) Acute effects of reactive oxygen and nitrogen species on the contractile function of skeletal muscle. J Physiol (Lond) 589: 2119–2127 doi:10.1113/jphysiol.2010.199059 21041533 10.1113/jphysiol.2010.199059 PMC3098691

[47] Greensmith DJ, Eisner DA, Nirmalan M (2010) The effects of hydrogen peroxide on intracellular calcium handling and contractility in the rat ventricular myocyte. Cell Calcium 48: 341–351 doi:10.1016/j.ceca.2010.10.007 21106236 10.1016/j.ceca.2010.10.007

