# Supplementary material for: Micro-Arrayed Human Embryonic Stem Cells-Derived Cardiomyocytes for In Vitro Functional Assay
Source: PLoS One. 2012 Nov 12;7(11):e48483. doi: 10.1371/journal.pone.0048483 (PMC3495940; doi:10.1371/journal.pone.0048483)
Supplement: Materials and Methods S1 — Supplementary information about hCMs culture medium, immunohystochemistry and Gap-FRAP analysis. (PDF) [file pone.0048483.s001.pdf]

**Micro-arrayed human embryonic stem cells-derived cardiomyocytes for *in vitro* functional assay**

Elena Serena, Elisa Cimetta, Susi Zatti, Tania Zaglia, Monica Zagallo, Gordon Keller, Nicola Elvassore

## Supplementary materials and methods

Basal medium

Stempro 34 (Invitrogen), 1% Penicillin/streptomicyn (Gibco), 1% L-Glutamine (Gibco), 150 µg/mL Transferrin (Roche), 50 ng/mL ascorbic acid (Sigma), 0.45 mM mono-Thioglycerol (MTG, Sigma Aldrich).

Immunohystochemistry

Cells were fixed with 2% PFA (Sigma Aldrich) for 7 min at room temperature, permeabilized with 0.5% Triton X-100 (Sigma-Aldrich) and blocked in PBS-2% horse serum (HS) for 45 min at room temperature. For Cx43 the fixation and permeabilization steps were performed in acetone for 10 minutes at -20°C. Primary antibodies were individually applied for 1 hour at 37°C. Cells were washed and incubated with Alexa488 and Alexa495 fluorescence-conjugated secondary antibody (Invitrogen) against mouse or goat for 45 min at 37°C. Nuclei were counterstained with hoechst (invitrogen), samples were mounted with Elvanol® and viewed under a fluorescence microscope (Leica, CTR6500).

Gap-FRAP analysis

In order to evaluate the functional interconnection between hCM, gap junction functionality was quantitatively determined in living cells by gap-FRAP assay. hMCs were loaded with calcein AM (3 µM, 45 min, Invitrogen). Due to its low molecular weights (622 Da), calcein AM has been shown to permeate gap junction channels. The cell culture was washed several times with PBS to remove the fluorochrome-ester and prevent further dye loading during subsequent measurements. FRAP was performed using a confocal laser scanning microscope (Leica), constituted by a reversed microscope equipped with an argon laser source at 496 nm. The intensity of the fluorescence signal was measured between 500 and 560 nm (emission band-pass filter) with a 20X objective and 5X optical zoom. The laser output power was adjusted using a powermeter to to 55 µW for 30 s to achieve sufficient photobleaching for fluorescence recovery observations, without causing visible damage. Fluorescence recovery analysis was performed for 450 s with a measurement every 10 s, with the laser output power being adjusted to 10 µW.

The fluorescence recovery kinetics was divided into several phases. First, the intensity of fluorescence measured in the target cell before photobleaching was recorded (F0). The fluorescence intensity value was measured at each time point during the recovery (F). For each experiment, this value was normalized by the intensity value of the reference cell (Fr) chosen at the edge of the microscopic field to account for possible photodegradation caused by the successive acquisitions and the leakage of the fluorescent dye. The ratio (F/Fr) allowed us to correct for changes in fluorescence intensity due primarily to photobleaching caused by exposure of the whole field to the excitation light source. The exponential fluorescence recovery as function of time was correlated by the following perturbation–relaxation equation:

$$\frac{F(t)}{F_r(t)} = A\left(1 - e^{-t/\tau}\right) \tag{1}$$

where $t$ is time [s] after photobleaching, $A$ is an adimensional parameter that sets the asymptotic level of recovery and $\tau$ the inverse value of transfer constant [s]. Values of fitting parameter were obtained from 20 replicates.

To exclude the eventuality of a recovery due to transplanar diffusion in the same cell, it has been necessary to evaluate fluorescence recovery in isolated calcein loaded cells.
