# Supplementary figures and images for: Micro-Arrayed Human Embryonic Stem Cells-Derived Cardiomyocytes for In Vitro Functional Assay
Source: PLoS One. 2012 Nov 12;7(11):e48483. doi: 10.1371/journal.pone.0048483 (PMC3495940; doi:10.1371/journal.pone.0048483)

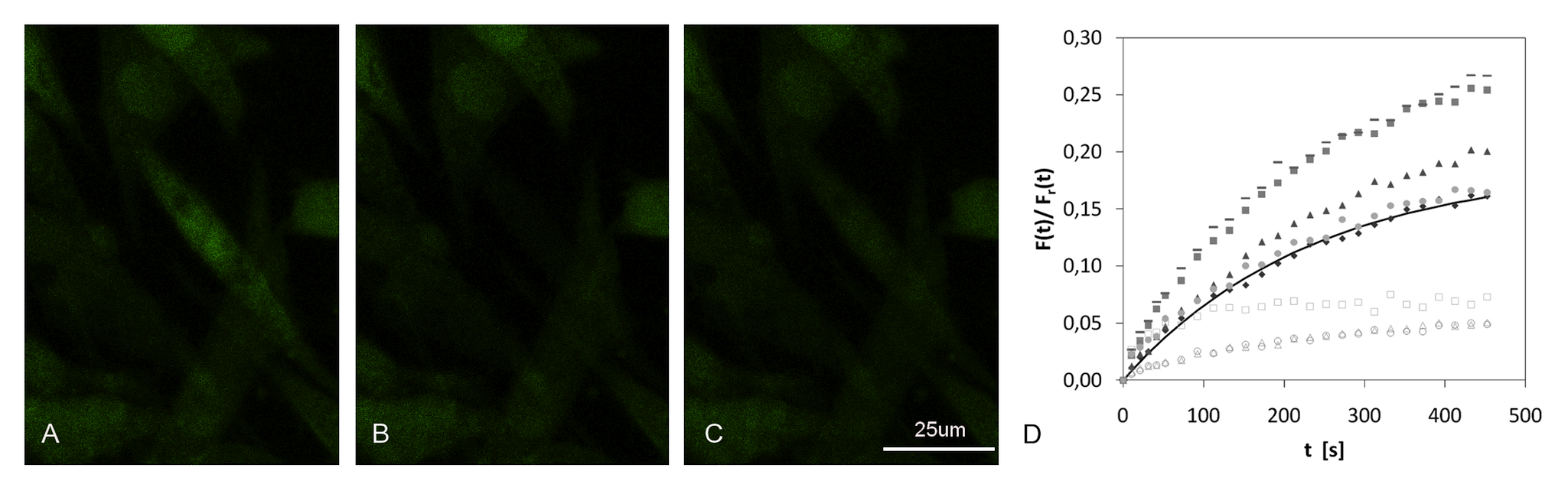

Supplement: Figure S1 — Gap-FRAP analysis. A–C - Representative images of fluorescence restoration in hCMs, target cell is indicated by an arrow, scale bar: 50 µm. A - Intensity of calceine AM fluorescence before photobleaching. B - Fluorescence right after photobleaching. C -Fluorescence recovery after 7.5 minutes. D - Graph representing the kinetic profiles of raw and fitted recovery data. (TIF) [file pone.0048483.s002.tif]

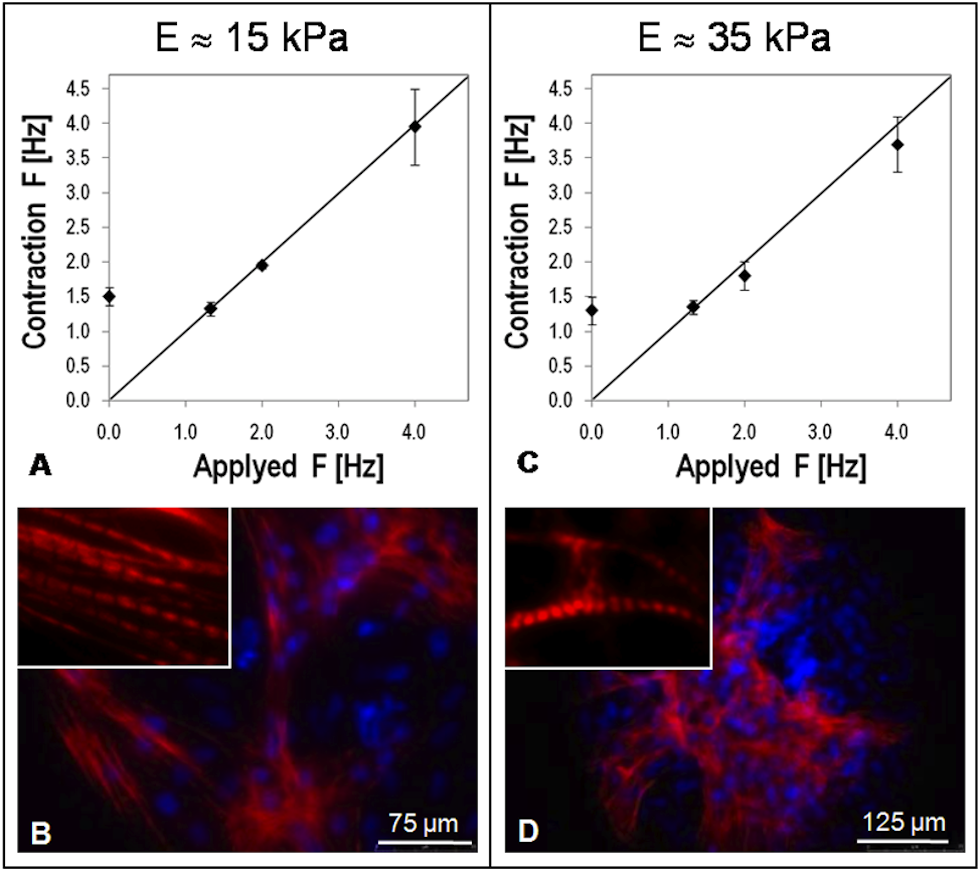

Supplement: Figure S2 — Effects of susbtrate stiffness on hCMs.A, B - Mophometric analysis and cTnT immunofluorescence of hCMs cultured onto 15 kPa. C, D - Mophometric analysis and cTnT immunofluorescence of hCMs cultured onto a 35 kPa hydrogel. Nuclei were counterstained with hoechst. (TIF) [file pone.0048483.s003.tif]
