# Supplementary material for: Micro-Arrayed Human Embryonic Stem Cells-Derived Cardiomyocytes for In Vitro Functional Assay
Source: PLoS One. 2012 Nov 12;7(11):e48483. doi: 10.1371/journal.pone.0048483 (PMC3495940; doi:10.1371/journal.pone.0048483)
Supplement: Table S1 — Gap-FRAP analysis. The table reports the value of A and k. (DOCX) [file pone.0048483.s005.docx]

**Table S1. Gap-FRAP analysis.**

| **Cell type** | **K [l/min]** | **A** |
| --- | --- | --- |
| hCMs | 0.34 ± 0.11 | 24.3 ± 5.5 % |
| Control | / | 5.5 ± 0.6 % |
